# Identification of miRNAs associated with *Aspergillus flavus* infection and their targets in groundnut (*Arachis hypogaea* L.)

**DOI:** 10.1186/s12870-025-06322-2

**Published:** 2025-03-18

**Authors:** Pushpesh Joshi, Vinay Sharma, Arun K. Pandey, Spurthi N. Nayak, Prasad Bajaj, Hari K. Sudini, Shailendra Sharma, Rajeev K. Varshney, Manish K. Pandey

**Affiliations:** 1 Center of Excellence in Genomics & Systems Biology (CEGSB), and Center for Pre-Breeding Research (CPBR), International Crops Research Institute for the Semi-Arid Tropics (ICRISAT), Hyderabad, India; 2 Department of Genetics and Plant Breeding, Chaudhary Charan Singh University, Meerut, India; 3 Department of Biotechnology, University of Agricultural Sciences, Dharwad, India; 4 Centre for Crop and Food Innovation, WA State Agricultural Biotechnology Centre, Murdoch University, Murdoch, Australia

**Keywords:** *Aspergillus flavus*, Differential expression, Genes, Groundnut, MicroRNA

## Abstract

**Background:**

The quality of groundnut produce is adversely impacted due to aflatoxin contamination by the fungus *Aspergillus flavus*. Although the transcriptomic control is not fully understood, the interaction between long non-coding RNAs and microRNAs in regulating *A. flavus* and aflatoxin contamination remains unclear. This study was carried out to identify microRNAs (miRNAs) to enhance the understanding of in vitro seed colonization (IVSC) resistance mechanism in groundnut.

**Result:**

In this study, resistant (J 11) and susceptible (JL 24) varieties of groundnut were treated with toxigenic *A. flavus* (strain AF-11–4), and total RNA was extracted at 1 day after inoculation (1 DAI), 2 DAI, 3 DAI and 7 DAI. Seeds of JL 24 showed higher mycelial growth than J 11 at successive days after inoculation. A total of 208 known miRNAs belonging to 36 miRNA families, with length varying from 20–24 nucleotides, were identified, along with 27 novel miRNAs, with length varying from 20–22 nucleotides. Using psRNATarget server, 952 targets were identified for all the miRNAs. The targeted genes function as disease resistant proteins encoding, auxin responsive proteins, squamosa promoter binding like proteins, transcription factors, pentatricopeptide repeat-containing proteins and growth regulating factors. Through differential expression analysis, seven miRNAs (aly-miR156d-3p, csi-miR1515a, gma-miR396e, mtr-miR2118, novo-miR-n27, ptc-miR482d-3p and ppe-miR396a) were found common among 1 DAI, 2 DAI, 3 DAI and 7 DAI in J 11, whereas ten miRNAs (csi-miR159a-5p, csi-miR164a-3p, novo-miR-n17, novo-miR-n2, osa-miR162b, mtr-miR2118, ptc-miR482d-3p, ptc-miR167f-3p, stu-miR319-3p and zma-miR396b-3p) were found common among 1 DAI, 2 DAI, 3 DAI and 7 DAI in JL 24. Two miRNAs, ptc-miR482d-3p and mtr-miR2118, showed contrasting expression at different time intervals between J 11 and JL 24. These two miRNAs were found to target those genes with NBS-LRR function, making them potential candidates for marker development in groundnut breeding programs aimed at enhancing resistance against *A. flavus* infection.

**Conclusion:**

This study enhances our understanding of the involvement of two miRNAs namely, ptc-miR482d-3p and mtr-miR2118, along with their NBS-LRR targets, in conferring resistance against *A. flavus*-induced aflatoxin contamination in groundnut under in vitro conditions.

**Supplementary Information:**

The online version contains supplementary material available at 10.1186/s12870-025-06322-2.

## Introduction

Groundnut (*Arachis hypogaea* L.) is an important legume crop that possesses abundant protein and oil content. It is cultivated across approximately 30.5 million hectares worldwide, resulting in an annual production of approximately 54.2 million tonnes [1]. The centre of origin of groundnut is reported in Gran Panatanal (Mato Grosso, Brazil) and on the eastern slopes of the Bovilian Andes [2]. It plays a vital role in enhancing food security by promoting nutrition in many developing countries [3]. Groundnut stands apart from other leguminous crops due to its unique characteristic of developing a gynophore that forms pods underground. The wild species of groundnut are diploids, whereas cultivated species are allotetraploid with 2n = 4x = 40, AABB-type genome. Groundnut breeding aims to achieve maximized yield, enhanced nutritional content, resistance to biotic and abiotic stresses, and ensuring compatibility with mechanized farming and harvesting practices. The yield of groundnut is severely affected by biotic and abiotic stresses. Among biotic stresses, aflatoxin contamination poses major pre- and post-harvest losses upto 13–59% worldwide [4]. Aflatoxins are carcinogenic mycotoxin with immunosuppressive properties produced by fungi genus *Aspergillus* [5]. Moreover, aflatoxin, B1, B2, G1, and G2 are most toxic mycotoxin naturally occurring in groundnut. These toxins hold greater significance compared to other fungal toxins due to their carcinogenic effects and potential for acute poisoning [6]. *A. flavus* is the most common species producing aflatoxin [7]. However, other species such as *A. parasiticus* and *A. nomius* might be source of contamination in certain regions [8, 9]. Aflatoxin came into focus in 1960s when large number of Turkey birds died in UK due to aflatoxin-contaminated feed. Apart from groundnut, these fungi also contaminate the other commodities such as rice, chilli pepper, wheat, maize and tree nut [10–12]. Chronic exposure to high level of aflatoxin has adverse impact on human and is considered a growth retardant factor in young individuals, and increasing the vulnerability to auto-immune deficiency symptoms [13, 14].

The fungus tends to invade groundnut crop at three stages: pre-harvest, during crop development and post-harvest stages [15, 16]. The fungal entry commonly occurs through the cracks that develop on the pod during pod maturation stage [17]. The severity of this infection increases when drought occurs, leading to inadequate water availability during pod development and resulting in the formation of cracks on the pod wall [18]. The *A. flavus* generally released the asexual spore namely, conidia [19]. The host–pathogen interaction of *A. flavus* is carried out by oxylipin-based signalling in fungus. These oxylipin-based products encoded by *psi-producing oxygenase* (*ppo*) gene have been reported to regulate the production of sclerotia, conidia and secondary metabolites in *A. flavus* [20–22]. The mycelia are produced saprophytically by the sclerotia, which serve as a resting stage for survival. These mycelia produce conidiophores which form conidia during favourable conditions (hot and dry weather) which were transported through wind, insects, pests, crop debris and infect economic parts of succeeding crop [19, 23]. Upon reaching the inside of the pod, *A. flavus* initiates to derive nutrients from the kernels and subsequently produce aflatoxin [24]. Developing groundnut varieties resistant to *A. flavus* infection is considered as an economically viable solution to mitigate aflatoxin contamination in regions where groundnuts are cultivated. However, this task presents several challenges for breeders. One major challenge is the lack of reliable resistance resources available. It requires significant time, effort, and financial investment to identify and introgress genomic regions that confer resistance to *A. flavus* infection into groundnut varieties. Another challenge lies in the complex and often hidden interactions between the plant and the fungus. The mechanisms by which groundnut plants resist or tolerate *A. flavus* infection are not fully understood, making it difficult to accurately select and breed for resistance traits. Furthermore, environmental factors play a crucial role in the development and spread of *A. flavus* infection. Temperature, humidity, soil conditions, and other environmental variables can influence the severity and prevalence of the fungus. Thus, these environmental effects must account when developing resistant varieties, adding another layer of complexity to the breeding process [25]. Groundnut is known to exhibit three resistance mechanism: namely resistance to in vitro seed colonization (IVSC), pre-harvest aflatoxin contamination and resistance to post-harvest aflatoxin production in seeds [26]. Investigating the molecular components of aflatoxin resistance is necessary to explore the origins of resistance through three distinct mechanisms. Unravelling the molecular mechanisms and identifying genes associated with IVSC resistance holds the promise of transforming the control of fungal colonization and aflatoxin contamination in groundnut. The progress made in genomics research has facilitated the sequencing of several groundnut genomes, including three cultivated allotetraploid varieties (Tiffrunner, Shitouqi, and Fuhuasheng) alongside two ancestral diploid species (*A. duranensis* and *A. ipaensis*) [27–30]. These sequencing efforts have opened up avenues for comprehensive explorations into the resistance against *A. flavus* infection, presenting new opportunities for in-depth investigations. However, several studies have been reported for breeding against aflatoxin contamination. The comparative transcriptome analysis and weighted gene co-expression network analysis were employed to investigate the resistance mechanism of groundnut against *A. flavus*. Their findings suggest that pathogenesis-related proteins, serine/threonine kinase, MAPK kinase, and pattern recognition receptors play crucial roles in groundnut’s ability to resist *A. flavus* [31].

The significance of the miRNAs in various biological processes such as counteracting environmental effects, developmental transitions, stabilizing genome and defesce responses against various pathogens, has been reported in eukaryotes [32]. The microRNAs (miRNAs) are small non-coding RNA comprised of 21–24 nucleotides (nt) present in both plants and animals. Historically, the first miRNA was Lin-4, identified in *Caenorhabditis elegans*, while the first miRNA in plant was discovered in *Arabidopsis* [33–35]. Moreover, the first miRNA in groundnut was discovered in 2010 using the high-throughput Solexa sequencing [36]. Several miRNAs have been discovered to control abiotic stress factors such as drought, salinity, cold, and heat, as well as biotic stress factors such as pathogenesis of bacteria, fungi, nematodes through post-transcriptional regulations [37, 38]. The significance of miRNAs in plant against biotic stress factors has been extensively explored. miRNAs exert their influence on target genes by binding specifically to targeting sites on gene transcripts. This sequence-specific binding can result in either degradation of the target mRNA or translational repression, mediated by proteins associated with the miRNA [39]. Moreover, miRNAs play critical role in leaf morphogenesis, floral development and root initiation and development [34]. Several known and novel miRNAs were identified that induced or inhibited upon infection by *Ralstonia solanacearum* through high-throughput genotyping in susceptible and resistant cultivars of groundnut [35]. The miRNA family (miR family) (miR2118) found to be associated with *NBS-LRR* gene whose expression was upregulated in resistant cultivar. The miRNAs, miR472/RDR6 proven to modulate PAMP-triggered immunity (PTI) and effector triggered immunity (ETI) through the post-transcriptional regulation in *Arabidopsis* [40]. The occurrence of multiple miRNAs in defense response against blast causing fungus *Magnaporthe oryzae* has already been reported in rice [41, 42]. With this background, we attempted to identify miRNAs to enhance the understanding of IVSC resistance mechanism in groundnut.

## Materials and methods

### Plant material, stress treatment and RNA extraction

The miRNA study aimed to examine the resistance to IVSC by using the resistant (J 11) and susceptible (JL 24) varieties of groundnut and a highly toxigenic strain of *A. flavus* (AF 11–4), which was identified by the Groundnut Pathology Unit of ICRISAT [43]. The strain was cultivated in a pure culture on Potato Dextrose Agar for seven days, after which a conidial suspension was prepared at a concentration of 10^6^ spores/ml.

### Screening of in vitro seed colonization

Surface sterilization of 100 healthy seeds each of the J 11 and JL 24 varieties were carried out using 0.1% HgCl_2_ for 3 min. Subsequently, the seeds underwent three rinses with sterile distilled water. For each variety, two distinct sets were made, comprising a control group and an infected group. Approximately 50 sterilized seeds of each variety were placed on sterile filter papers in petri dishes to serve as control. The other 50 seeds were immersed in 40.0 ml spore suspension of the toxigenic strain ‘AF 11–4’ of *A. flavus* at an optimal concentration of 10^6^ spores/ml for 4 min. Both the sets were incubated in a dark, and humid chamber at temperature of 28 °C with 100% relative humidity. RNA samples were collected from both the infected and control groups of J 11 and JL 24 varieties at 1 day, 2 days, 3 days, and 7 days after inoculation (1 DAI, 2 DAI, 3 DAI, and 7 DAI). During each time interval, a few seeds were used for the microscopic examination of the seed coat and for estimating the aflatoxin level. The experiment was carried out twice, and each set was considered as a separate biological replicate. To estimate the aflatoxin concentration, 16 samples were analysed, consisting of two varieties, four stages, and two treatments.

### Aflatoxin quantification and microscopic observation of seed coat

The quantitative estimation of total aflatoxins accumulated under both the control and infected treatments was carried out using an indirect competitive enzyme-linked immunosorbent assay (ELISA). The assay employed polyclonal antibodies produced against Aflatoxin B1 (AFB1) as explained by Waliyar et al. [16]. The seed coats of both infected and control varieties were observed under a stereomicroscope.

### RNA isolation and sequencing

The “NucleoSpin® RNA Plant” kit (Macherey–Nagel, Germany) was utilized to isolate total RNA from the seeds. The quality and quantity of RNA were assessed using a Nanodrop 1000 spectrophotometer (Thermo Fisher Scientific Inc, USA). For the construction of the cDNA library, approximately 5 μg of total RNA was used, which was pooled together in equal quantities from two biological replicates. RNA samples that were sequenced on the Illumina HiSeq 2500 platform met the following quality criteria: a 260/280 ratio between 1.8 to 2.1, a 260/230 ratio between 2.0 to 2.3, and a RIN (RNA integrity number) value greater than 7.0. The paired-end reads of 2 × 100 bp were generated from the samples, and following a quality control (QC) analysis with NGS-QC box, filtered reads were obtained.

### miRNA sequencing and data pre-processing

The Illumina TruSeq Small RNA Library Prep Kit (Illumina Inc., San Diego, CA) was used to construct the small RNA libraries according to the manufacturer’s instructions. After separating 1 µg of RNA from each sample using polyacrylamide gel electrophoresis (PAGE), 18–30 nt long RNA fragments were enriched, followed by ligation of 3' and 5' adapter using T4 RNA ligase.

After adapter ligation of RNA molecules, cDNA was synthesized, amplified, and subsequently sequenced on the Illumina HiSeq 4000. “Trimmomatic v0.35” was used to perform several quality control steps on the raw reads obtained from sequencing, including the removal of low-quality reads, reads with adapter or primer contamination, and those with a poly-A tail. Reads shorter than 18 nt and longer than 35 nt were rejected. After obtaining clean reads from each sample, these were subjected to additional screening to remove any rRNA, tRNA, snoRNA, or repeat sequences. Once the filtering was done, the repeated reads were converted into distinct sequences, which were assigned read counts to facilitate miRNA prediction.

### Identification of known and novel miRNA

Conserved miRNAs were identified using the miRbase [44] by mapping filtered unique reads of each sample onto plant miRNAs. The alignment procedure involved using the Bowtie alignment tool v1.1.2 [45] with a tolerance of two mismatches. Any unaligned unique reads were subsequently subjected to novel miRNA prediction. The unique reads that remained were mapped onto the groundnut genome using the Bowtie, with no allowance for mismatches. Subsequently, putative precursor sequences, spanning 250 bp, were extracted for the aligned reads. Using the miRDeep-P, a probabilistic model-based software, novel miRNAs were identified from the identified precursor sequences [46]. MiRDeep-P introduces a novel prediction approach that takes into account various factors including the secondary structure, the presence of a 3'-overhang, evidence of star miRNA, the length difference between mature and star miRNA (which should be less than six nucleotides), the Dicer cleavage site, and the minimum free energy of the small RNA reads [47]. Moreover, the miRNAs that were identified were grouped into families using CD-HIT [47] with a 90% identity threshold, based on their sequence similarity. Afterwards, the psRNATarget server [48] was utilized with default parameters to predict the mRNA targets of the identified miRNAs.

### Expression analysis of miRNAs

To evaluate miRNA expression levels and normalize raw reads count, DESeq2 was used [49]. A miRNA was considered significantly expressed if it possessed log2 fold change ≥ 1 or ≤ −1 and a *P*-*value* ≤ 0.05.

### Genes expression

The *A. hypogaea* gene expression atlas (AhGEA) specific to the *fastigiata* sub-species (BioProject ID: PRJNA484860) was utilized to examine the tissue-specific expression patterns of the selected genes [50]. The expression trends of targeted genes were clustered using a package “Mfuzz version 3.19” in R-studio [51].

## Results

### Microscopic observation and aflatoxin estimation

**Fig. 1:**
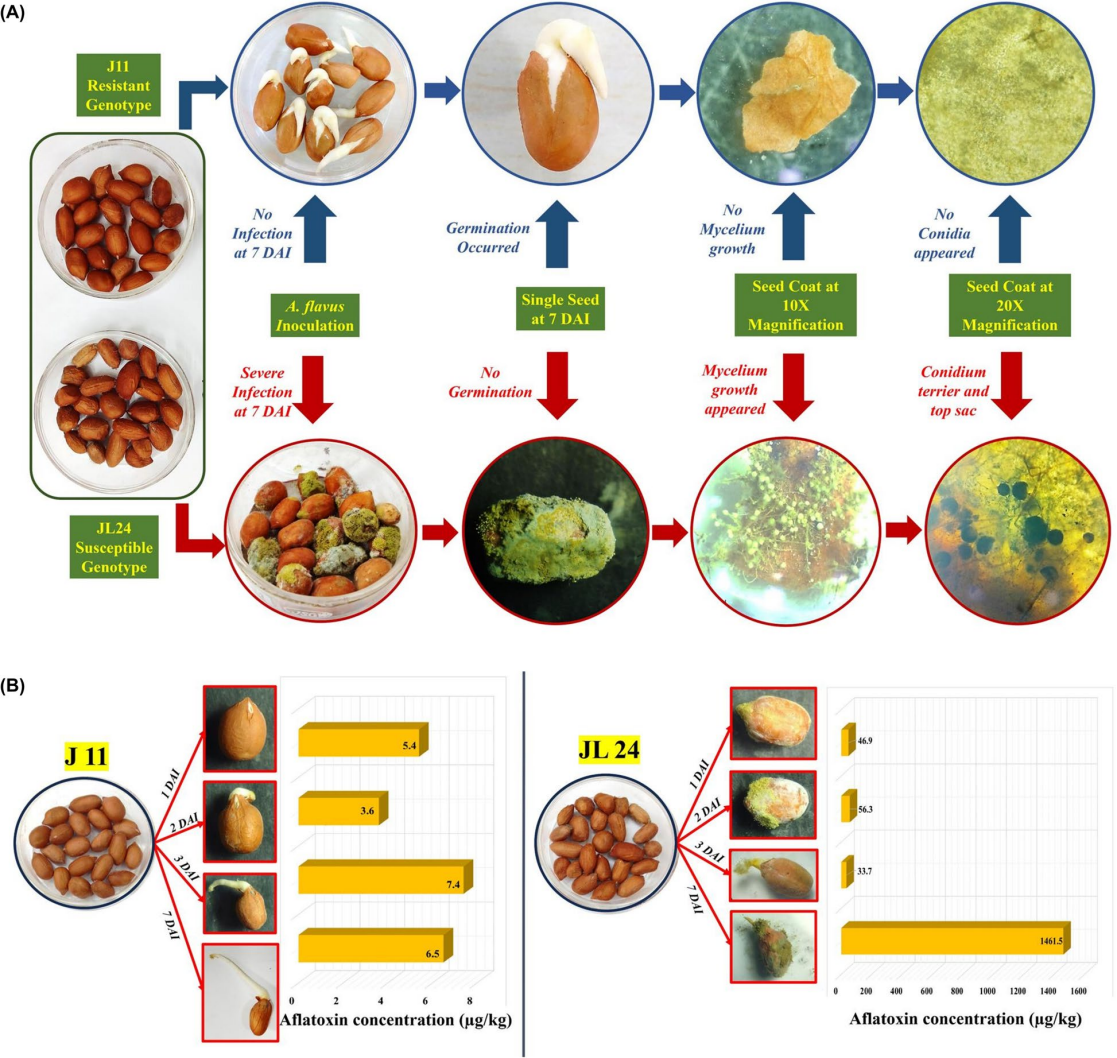
Microscopic observations and aflatoxin quantification of J 11 and JL 24; **A** Growth of mycelia on seed coat after 7^th^ day of inoculation in susceptible variety (JL 24) and resistant variety (J 11);** B** Phenotypic observations of seeds of J 11 and JL 24 and graphical representation of aflatoxin contamination at different time points (1 DAI, 2 DAI, 3 DAI and 7 DAI)

Using stereomicroscope, mycelial growth was seen considerably low at first day after inoculation (1 DAI) in both J 11 and JL 24 varieties. The seeds of JL 24 showed higher mycelial growth than J 11 at 2 DAI and at subsequent periods (3 DAI and 7 DAI). The variety J 11 showed no fungal colonization, whereas JL 24 noticed with considerable colonization. The germination of both the varieties was uniform at controlled conditions. However, the seeds JL 24 couldn’t germinate after inoculation due to heavy fungal growth and colonization (Fig. 1(A)).

### High-throughput miRNA sequencing

The resistant (J 11) and susceptible (JL 24) varieties exhibited significant difference in the aflatoxin content upon *A. flavus* infection on seeds (Fig. 1B). To study the variations of miRNAs during *A. flavus* infection in seeds, four inoculation periods were selected: namely, 1 day after inoculation (1 DAI), 2 days after inoculation (2 DAI), 3 days after inoculation (3 DAI) and 7 days after inoculation (7 DAI). In this way, 4 infected days (ID) and 4 controlled days (CD) for J 11 and JL 24 were included in the experiment. A total of 16 small RNA libraries were constructed from aflatoxin infected seeds (J 11_ID1, J 11_ID2, J 11_ID3, J 11_ID7, JL 24_ID1, JL 24_ID2, JL 24_ID3, JL 24_ID7) and seeds at controlled condition (J 11_CD1, J 11_CD2, J 11_CD3, J 11_CD7, JL 24_CD1, JL 24_CD2, JL 24_CD3, JL 24_CD7) and sequenced using the Illumina/Solexa 500 platform to identify the aflatoxin-related miRNA in groundnut. Total of 782.65 million reads were generated with an average of 48.91 million reads per sample (Table 1). After subsequent steps of filtering low quality reads and trimming, a set of 543.85 million high quality reads was retained for further analysis. Around 509.36 million clean reads with length 15-nt ≤ reads ≤ 30-nt were obtained.

**Table 1:** Statistical analysis of small RNAs mapped in groundnut genome

Treatment	RW	QFR	FN	15 ≤ Reads ≤ 30 nt	FRNC	FC	FR	FER
J 11-CD1	54,733,510	38,290,230	38,289,935	37,607,501	2,773,759	2,730,265	2,307,710	1,282,733
J 11-CD2	34,160,093	25,581,901	25,581,710	24,315,116	2,212,974	2,180,634	1,855,181	1,156,020
J 11-CD3	38,339,938	32,633,469	32,633,132	24,721,003	4,916,621	4,829,220	4,135,336	2,422,427
J 11-CD7	25,631,514	15,375,955	15,375,860	15,073,897	740,328	725,596	608,319	381,023
JL 24-CD1	37,556,017	31,438,065	31,437,804	28,912,948	1,826,128	1,807,981	1,571,559	931,563
JL 24-CD2	96,122,457	63,723,184	63,722,354	60,796,033	15,275,895	14,849,698	11,950,811	7,234,406
JL 24-CD3	70,078,765	57,731,276	57,731,226	53,277,043	6,723,254	6,616,518	5,577,768	3,729,203
JL 24-CD7	34,872,344	27,533,632	27,533,600	25,547,125	3,754,098	3,678,154	3,207,151	2,160,496
J 11-ID1	43,370,573	27,052,365	27,052,357	26,588,590	1,055,798	1,042,247	870,828	475,804
J 11-ID2	36,088,471	20,740,745	20,740,319	18,139,228	4,378,778	4,294,535	3,516,043	2,122,107
J 11-ID3	34,220,378	20,622,878	20,622,349	16,947,807	3,988,497	3,905,385	3,213,088	1,868,655
J 11-ID7	52,216,928	35,637,927	35,637,918	34,723,693	2,803,845	2,749,333	2,244,547	1,323,987
JL 24-ID1	102,952,235	68,520,018	68,519,053	65,035,009	8,681,231	8,536,505	6,770,784	3,587,796
JL 24-ID2	39,858,694	26,175,601	26,175,592	25,559,301	2,868,239	2,841,539	2,340,381	1,388,715
JL 24-ID3	47,355,756	30,784,013	30,784,006	30,363,484	2,405,071	2,375,130	1,864,742	1,097,799
JL 24-ID7	35,093,653	22,009,701	22,009,696	21,759,049	2,868,239	2,841,539	2,340,381	1,388,715
**Total**	**782,651,326**	**543,850,960**		**509,366,827**				

*RW* Raw reads, *QFR* Quality filtered reads, *FN* Filtered for Ns, *FRNC* Filtered reads for ncRNA, *FC* Filtered for chloroplast, *FR* Filtered for repeats, *FER* Filtered for exonic region, *CD* Controlled day, *ID* Infected day

**Fig. 2:**
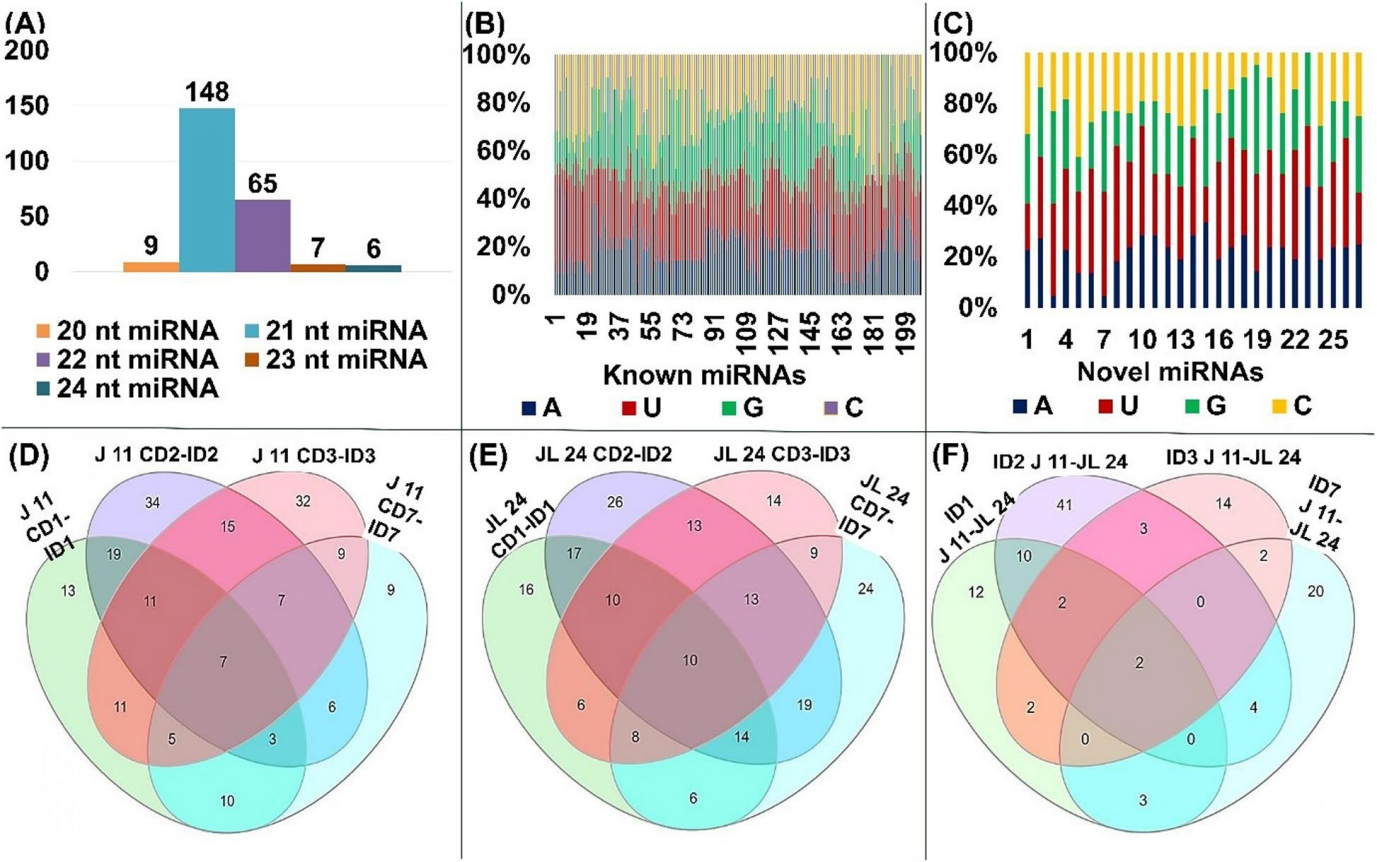
Total counts of genome-wide miRNAs;** A** Length distribution of miRNAs;** B** Relative proportion of bases in known miRNAs;** C** Relative proportion of bases in novel miRNAs;** D** Number of differentially expressed miRNAs between controlled and infected days in J 11; **E** Differentially expressed miRNAs between controlled and infected days in JL 24; **F** Differentially expressed miRNAs between J 11 and JL 24 at different days after inoculation

The length distribution of unique miRNA indicated that 21 nt (62.97%) were the most abundant class followed by 22-nt (27.65%), 20-nt (3.82%), 23-nt (2.97%), and 24-nt (2.55%) (Fig. 2A). The length of miRNAs within the range of 20–24 nt is in line of DCL cleaved product [34]. Most of the miRNA sequences, especially of 20-nt, 21-nt, 22-nt and 23-nt length, initiated with uridine (U). The 24-nt long miRNAs were adenine (A) as first nucleotide at 5’ end (Fig. 2B and C).

### Identification of known and novel miRNA

The miRNAs are known to play critical roles in response to both biotic and abiotic stresses. To identify known and novel miRNAs, filtered reads were mapped against miRNAs of related species through miRBase. A total of 50.9 million reads were mapped to miRBase, enabling the identification of 208 known miRNAs belonging to 36 miR families (Additional file 1). In addition to known miRNAs, plants also possess unique miRNAs for which unmapped reads were subjected to miRNA prediction processed through miRDeep-P. Briefly, mapped reads were used to obtain precursor sequences, which folded into possible stem-loop structures using the “Vienna” package and further filtered and processed. A total of 27 potential novel miRNAs with length ranged from 20 to 22 nt were obtained after the removal of those miRNA which could not meet the miRNA criteria. The average GC content of groundnut miRNAs was found to be 51.05%, similar to chickpea (48%) and soybean (46%). The known miRNAs were grouped into 36 families based on similarity-based clustering. Among these, miR166 was the largest family with 28 miRNA members, followed by miR156 (24 members) and miR167 (23 members). However, novel miRNAs could not fit into any of the conserved miRNA families.

### Differentially expressed miRNAs during *A. flavus* infection in groundnut seed

To identify differentially expressed miRNAs, expression patterns of identified miRNAs were evaluated in all libraries. The majority were found in more than one sample. Using the criteria of an adjusted *P*-*value* < 0.05 and fold change < −1 and > 1, differentially expressed miRNAs were identified. In variety J 11, we found 79, 102, 97, and 56 differentially expressed miRNAs between the controlled and infected samples at 1 DAI, 2 DAI, 3 DAI and 7 DAI, respectively. Among these, seven miRNAs, namely aly-miR156d-3p, csi-miR1515a, gma-miR396e, mtr-miR2118, novo-miR-n27, ptc-miR482d-3p, and ppe-miR396a, were consistently differentially expressed in the resistant variety at 1 DAI, 2 DAI, 3 DAI and 7 DAI. These seven miRNAs accounted for 3.6% of the total differentially expressed miRNAs in variety J 11. Between controlled and infected samples, the expression patterns of miRNAs from various families exhibited a combination of upregulation and downregulation at 1 DAI (Additional file 2). The miRNAs from the families such as miR159, miR160, miR166, miR167, miR171, miR319, and miR398 demonstrated both upregulation and downregulation. Conversely, families including miR156, miR1515, and miR162 showed upregulation, while miRNAs from the families miR168, miR169, miR408, miR395, miR396, and miR482 exhibited downregulation.

Among the novel miRNAs, majorly were upregulated except novo-miR-n17. In 2 DAI, the miRNAs from the families miR156, miR159, miR166, miR167, miR171, miR319 and miR482 showed both upregulation and downregulation. The miR families such as miR1507, miR1511, miR162, miR170, miR398, miR396 and miR408 were upregulated whereas miR1515, miR169, miR2118 and miR2199 displayed downregulation. In 3 DAI, the miR families namely, miR1511, miR1515, miR160, miR3509, miR3517 and miR3518 were upregulated whereas miR159, miR162, miR164, miR170, miR171, miR2118, miR319, miR396 and miR398 were downregulated. The miR families in which miRNAs exhibited upregulation as well as downregulation were miR156, miR166, miR167, miR168 and miR3509. Among the novel miRNAs, only three families (novo-miR-n3, novo-miR-n5 and novo-miR-n26) were downregulated. At 7 DAI, the miRNAs of miR156, miR159, miR166, miR168, miR319, miR396, miR398 and miR482 families, demonstrated both upregulation and downregulation. Majority of novel miRNAs were upregulated except novo-miR-n5.

In variety JL 24, differential expression of miRNAs was observed, with 87, 122, 83, and 103 miRNAs being differentially expressed between the controlled and infected conditions at 1 DAI, 2 DAI, 3 DAI and 7 DAI, respectively. Among these, a total of ten miRNAs, namely csi-miR159a-5p, csi-miR164a-3p, novo-miR-n17, novo-miR-n2, osa-miR162b, mtr-miR2118, ptc-miR482d-3p, ptc-miR167f-3p, stu-miR319-3p, and zma-miR396b-3p, were consistently differentially expressed in the susceptible variety at 1 DAI, 2 DAI, 3 DAI and 7 DAI. These ten miRNAs accounted for 4.8% of the differentially expressed miRNAs in JL 24. Between controlled and infected samples, the miRNAs from the families miR1507, miR1511, miR160, miR164, miR2118, miR3509 and miR479, were upregulated whereas miR families namely, miR3514 and miR390 were downregulated at 1 DAI. In contrast, the members of miR156, miR159, miR162, miR166, miR167, miR168, miR171, miR319, miR396 and miR482 displayed both upregulation and downregulation expression. At 2 DAI, miRNAs from the families such as miR156, miR159, miR166, miR171, miR390, miR396, and miR482 exhibited a combination of upregulation and downregulation. There were more families demonstrating upregulation than families showing downregulation. At 3DAI, the few miRNA families namely, miR156, miR159, miR160, miR166, miR167 and miR396 showed upregulation as well as downregulation. Among the novel miRNAs, only three miR families (novo-miR-n10, novo-miR-n17 and novo-miR-n6) were downregulated between controlled and infected samples. The miRNAs from the families miR156, miR160, miR162, miR167, miR171, miR2118, miR472 and miR482 were upregulated between controlled and infected samples at 7 DAI. Among the novel miRNA families, only three families, namely novo-miR-n2, novo-miR-n15, and novo-miR-n19, exhibited upregulation.

Between varieties J 11 and JL 24, a total of 31, 62, 25, and 37 miRNAs were observed as differentially expressed at 1 DAI, 2 DAI, 3 DAI and 7 DAI, respectively (as shown in Fig. 2(D, E, F)). Among these, two miRNAs, namely mtr-miR2118 and ptc-miR482d-3p, were consistently differentially expressed in both J 11 and JL 24 at 1 DAI, 2 DAI, 3 DAI and 7 DAI. These two miRNAs accounted for only 0.017% of the total differentially expressed miRNAs. Upon comparison between controlled and infected samples, the numbers of upregulated miRNAs were more than of downregulated miRNAs in J 11 and JL 24 (Table 2) (Additional file 2).

**Table 2:** Summary of numbers of down and up-regulated miRNAs in different combinations

Varieties	Treatments	No. of downregulated miRNAs	No. of upregulated miRNAs
J 11	CD1-ID1	46	33
J 11	CD2-ID2	41	61
J 11	CD3-ID3	53	44
J 11	CD7-ID7	25	31
**Total**		165	169
JL 24	CD1-ID1	33	54
JL 24	CD2-ID2	64	58
JL 24	CD3-ID3	37	46
JL 24	CD7-ID7	36	67
**Total**		170	225

*CD1* Controlled day 1, *ID1* Infected day 1, *CD2* Controlled day 2, *ID2* Infected day 2, *CD3* Controlled day 3, *ID3* Infected day 3, *CD7* Controlled day 7, *ID7* Infected day 7

### In silico target identification and gene ontology

**Fig. 3:**
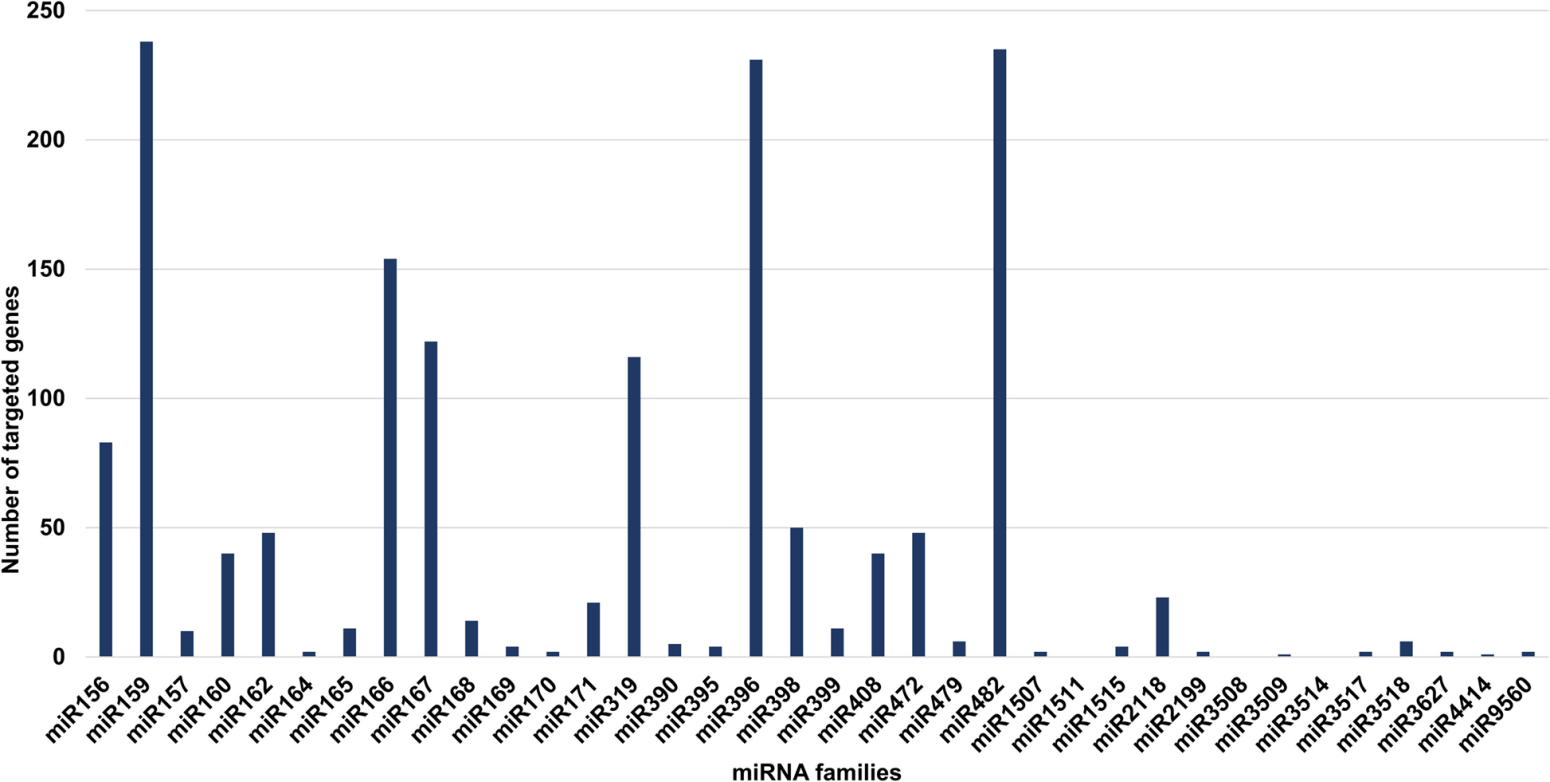
Graphical representation of number of targets from different miRNA families

Identification of target of miRNAs was conducted using psRNATarget server, based on complementarity between miRNAs and target sequence. Total 952 unique targeted genes were identified for 197 miRNAs. Among 952, 854 and 98 unique genes were found associated with 179 known and 18 novel miRNAs, respectively. Unfortunately, no targets were identified for 29 known and 9 novel miRNAs. Notably, there were instances where multiple miRNAs targeted the same genes, indicating a degree of overlap in their regulatory functions. This revealed that, 197 miRNAs (83.8%) exhibited targeting activity, resulting in the regulation of 1,742 genes. Among the 1,742 targeted genes, 1,643 genes were found to be targeted by known miRNAs, while the remaining 99 genes were targeted by novel miRNAs. The maximum targets were found for the members of family miR159 (238), followed by miR482 (235) and miR396 (231) (Fig. 3).

**Fig. 4:**
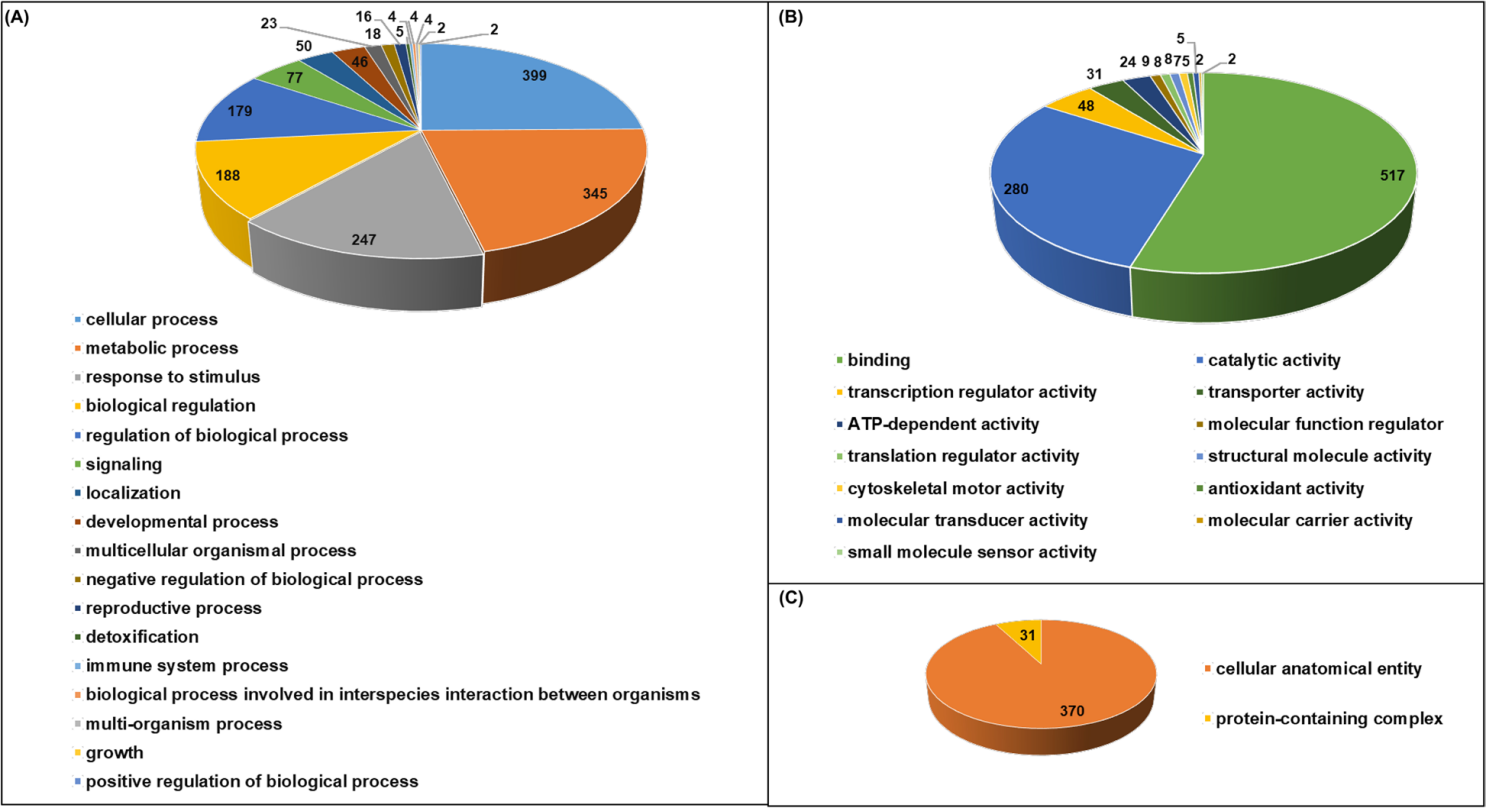
Gene Ontology enrichment analysis of all identified differentially expressed miRNAs involved in (**A**) Biological processes; **B** Molecular processes;** C** Cellular components

The target annotations grouped the genes into several categories, including disease resistance genes, cellular enzymes (kinase, methyltransferase, and β-galactosidase), transcription factors, proteasome assembly, proton transmembrane transport, meristem development and maintenance. The majority of annotated genes were responsible for disease resistance protein (22.8%), followed by transcription factors (3.6%), auxin responsive protein coding genes (3.5%), and pentatricopeptide repeat protein (3.5%). To understand the possible involvement of miRNA targets in the groundnut’s response to aflatoxin stress, a Gene Ontology (GO) enrichment analysis was carried out. A total of 1609 biological processes, 401 cellular components, and 946 molecular functions were allocated uniformly among the targets. Among biological process, the most significant GO terms were cellular process, metabolic process, response to stimulus, and biological regulations. Similarly, binding has most significant GO term followed by catalytic activity and transcription regulator activity among molecular function. In cellular component category, cellular anatomical entity has maximum GO term followed by protein-containing complex (Fig. 4).

### Common miRNA in two groundnut varieties at 1 DAI, 2 DAI, 3 DAI and 7 DAI

On comparing different days after inoculation for the same variety, we found more miRNAs were expressed at 2 DAI in J 11 as well as in JL 24. This indicated that the two varieties responded in more similar manner at 2 DAI. However, slope of number of miRNAs was raised at 7 DAI in JL 24 comparable to J 11 where slope diminished after 2 DAI. This showed the both varieties had started to respond to *A. flavus* infection from 2 DAI but susceptible variety showed different response at 7 DAI. Large number of differentially expressed common miRNAs were observed in both J 11 and JL 24 at 1 DAI, 2 DAI, 3 DAI and 7 DAI.

**Fig. 5:**
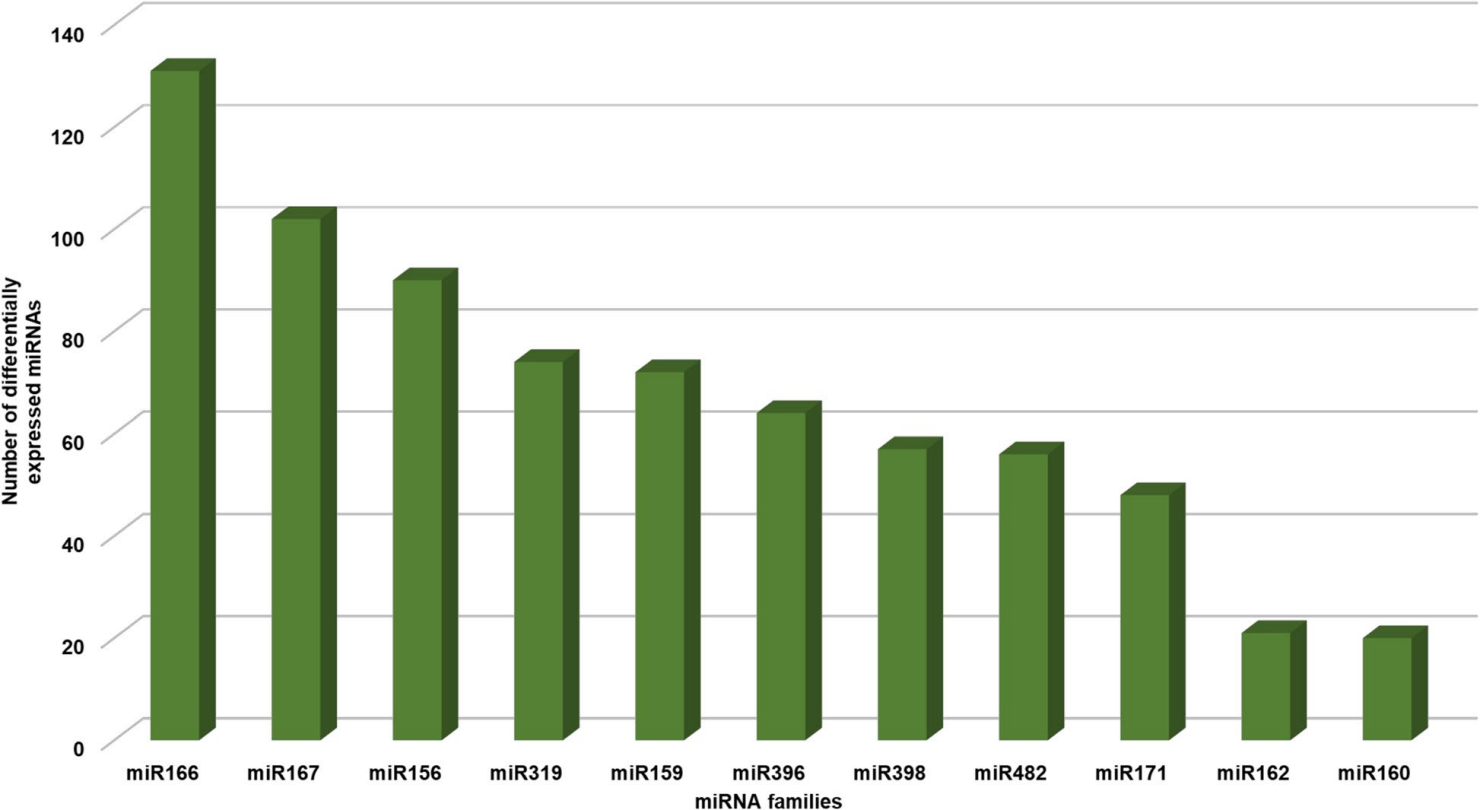
Graphical representation of numbers of differentially expressed miRNAs from different miR families

**Fig. 6:**
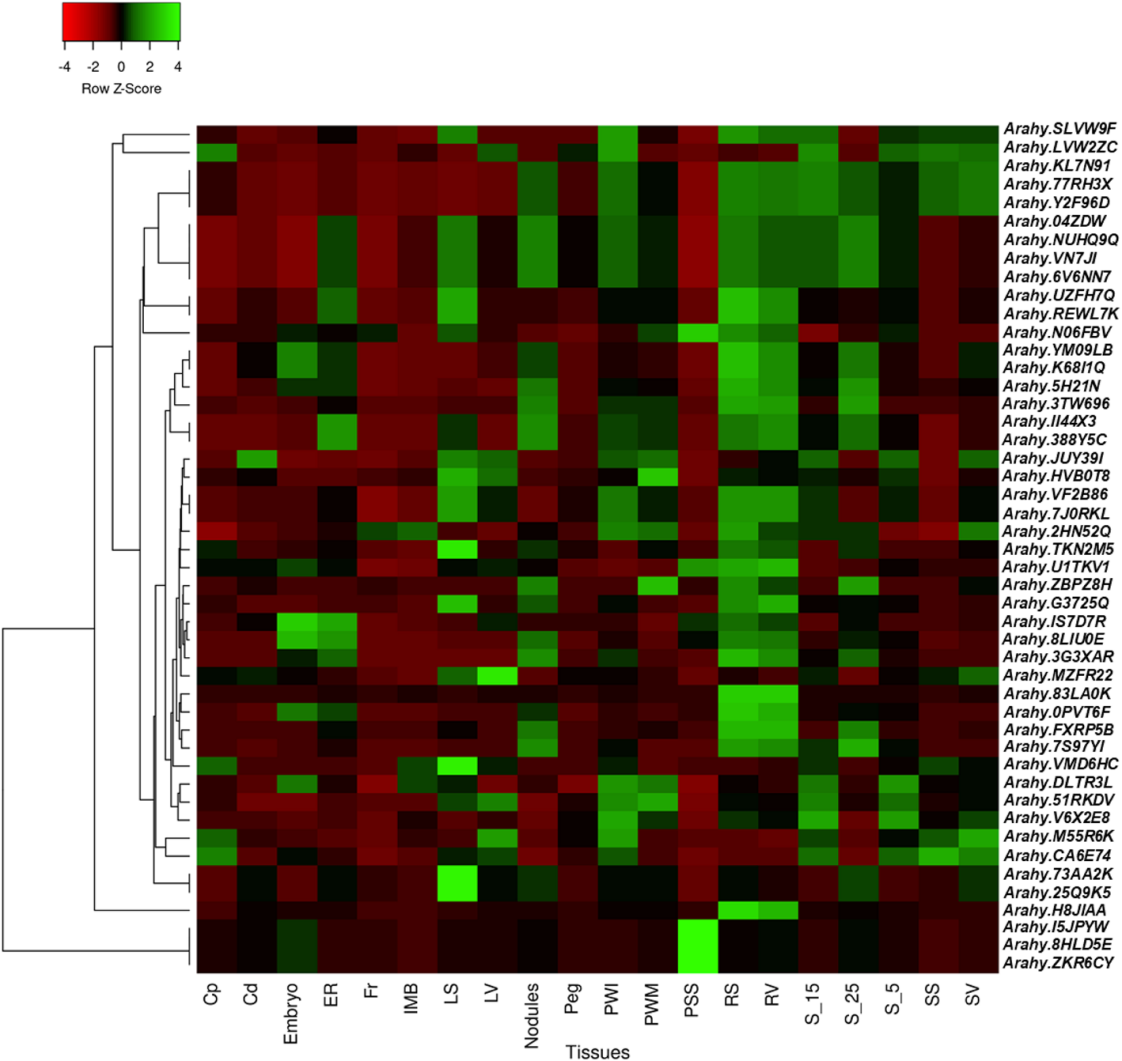
Tissue-specific expression pattern of targeted 47 genes of miRNAs (43 for miR-482d-3p and four for mtr-miR2118) across 20 different tissues; Cp: Coleoptile, Cd: Cotyledon; ER: Emerging radicle; Fr: Flower; IMB: Immature bud; LS: Leaves senescence; LV: Leaves vegetative; PWI: Pod wall immature; PWM: Pod wall mature; PSS: Pre-soaked seeds; RS: Root seedling; RV: Root vegetative; S_15: Seeds 15; S_25: Seeds 25; S_5: Seeds 5; SS: Shoot seedling; SV: Stem vegetative

Total 31 differentially expressed miRNAs were common between J 11 and JL 24 at 1 DAI. Among them, 11 miRNAs showing contrasting expression between J 11 and JL 24. Sixty-two differentially expressed miRNAs were common between J 11 and JL 24 at 2 DAI. Among them, 50 miRNAs showed contrasting expression between J 11 and JL 24. Similarly, 16 from 25 miRNAs and 16 from 31 miRNAs were showing contrasting expression between J 11 and JL 24 at 3 DAI and 7 DAI, respectively. The known miR families such as miR166, miR167 and miR156 were identified more frequently and abundantly expressed, consistent with previous studies (Fig. 5). To identify the miRNAs involved in aflatoxin resistance, we examined those common differentially expressed miRNAs which have contrasting expression patterns in resistant variety under 1 DAI, 2 DAI, 3 DAI and 7 DAI. Interestingly, two miRNAs namely mtr-miR2118 and ptc-miR482d-3p from miR2118 and miR482 families, respectively, were showed contrasting expression pattern between J 11 and JL 24 at all the infected days. Both miRNAs, ptc-miR482d-3p and mtr-miR-2118 showed downregulation in resistant variety, J 11 whereas it become upregulated in susceptible variety, JL 24 (Table 3). However, miRNAs such as zma-miR396b-3p and osa-miR162b also showed upregulation in JL 24 but didn’t express in J 11. These miRNAs were further examined for targeted genes, there were seven genes for mtr-miR2118 and 62 targets for ptc-miR482d-3p. The annotation of these targeted genes was found to be associated with disease resistance mechanism. For zma-miR396b-3p, six targets were identified, linked with LRR receptor-like serine/threonine-protein kinase metabolism. Similarly, osa-miR162b had 23 targeted genes associated with metabolism of beta amylase, UDP-glycosyltransferase, endoribonuclease Dicer homolog and pentatricopeptide repeat-containing proteins. The gene expression atlas (AhGEA) of *A. hypogaea* ssp. *fastigiata* was utilized to detect the expression of genes specific to certain tissues [40]. For mtr-miR2118, the expression of only four genes were found in different tissues in AhGEA. These genes were found in chromosome 9, 19, 5 and 15. Similarly, total 43 genes showed tissue specific expression associated with ptc-miR482d-3p in AhGEA. Among 43, maximum genes (21) were found on chromosome 12 followed by chromosome 2 (13 genes), chromosome 14 (4 genes), chromosome 4 (4 genes) and chromosome 3 (1 gene) (Table 4). The insilico expression of these genes using AhGEA were shown in Fig. 6 and Additional file 3. The expression trends of 47 genes were showed in total nine clusters (Additional file 4). Among these clusters, Cluster 1 consisted of four genes that showed upregulation in tissues of pre-soaked seeds. These genes were found to be involved in encoding the LRR and NB-ARC disease resistance-domains. In contrast to cluster 1, the cluster 2 consisted of three genes which showed downregulation in the tissues of pre-soaked seeds. The cluster 3 comprised six genes that exhibited upregulation in tissues such as leaves, immature pod walls, and roots of seedlings. In the cluster 4, the four genes showed similar trend of both upregulation and downregulation. Cluster 5 comprised nine genes that exhibited a consistent expression pattern across all tissues. In Cluster 6, all six genes showed downregulation specifically in the tissues of pre-soaked seeds. Cluster 7 consisted of four genes that were upregulated during the senescence phase of leaves. Additionally, Cluster 8 included five genes that showed upregulation in both immature and mature pod walls. Similarly, Cluster 9 consisted of six genes that exhibited a similar trend of both upregulation and downregulation across all 20 tissues. Importantly, all 47 genes were involved in mechanism of encoding disease resistance protein domains (TIR-NBS-LRR and LRR and NB-ARC domain).

**Table 3:** Differential expression of miRNAs common among 1 DAI, 2 DAI, 3 DAI and 7 DAI in J 11 and JL 24

Varieties	Differential Expression Pattern				
	**miRNAs**	**1 DAI**	**2 DAI**	**3 DAI**	**7 DAI**
**J 11**	aly-miR156d-3p	1.2333638	−1.904056	2.1020817	2.507183
	csi-miR1515a	2.7652149	−1.9477172	1.313454	1.1852549
	gma-miR396e	−2.1416757	1.1874424	−3.5345429	−1.263646
	mtr-miR2118	1.0282493	−1.9824826	−2.6600738	−3.0291357
	novo-miR-n27	1.7720686	−1.1817914	1.5628696	1.2441486
	ppe-miR396a	−1.9717507	1.3534523	−3.4090121	−1.8653712
	ptc-miR482d-3p	−2.1416757	−1.6160782	−1.7824791	−1.8851344
**JL 24**	csi-miR159a-5p	1.4456028	1.8431715	2.9909445	3.1198553
	csi-miR164a-3p	1.7575468	−2.2442914	2.0200909	1.1666936
	novo-miR-n17	−1.2676821	1.2468739	−1.2670718	−2.1762961
	novo-miR-n2	1.1136906	5.7691709	1.0030173	4.0842314
	osa-miR162b	1.0026593	1.2092994	1.0315865	2.821197
	ptc-miR167f-3p	1.5876218	3.4908697	1.1146716	3.2541564
	stu-miR319-3p	2.1725843	−1.418384	2.3671895	−1.2011708
	zma-miR396b-3p	2.1725843	1.1062059	1.5175905	3.7597965
	ptc-miR482d-3p	−1.8048254	−1.0048254	2.7943508	4.6891405
	mtr-miR2118	1.3013652	1.258209	1.0253275	2.8391189

*1 DAI* first day after inoculation, *2 DAI* second day after inoculation, *3 DAI* third day after inoculation, *7 DAI* seventh day after inoculation

**Table 4:** Summary of targeted 47 genes of miRNAs

miRNA	Targeted Gene model	Chromosome	Start position	End position	Annotations
miR2118	*Arahy.H8JIAA.1*	15	154,617,270	154,625,699	Disease resistance protein (TIR-NBS-LRR class) family
	*Arahy.7J0RKL.1*	9	6,874,895	6,880,710	Disease resistance protein (TIR-NBS-LRR class) family
	*Arahy.VF2B86.1*	19	8,244,428	8,250,223	Disease resistance protein (TIR-NBS-LRR class) family
	*Arahy.HVB0T8.1*	5	90,137,426	90,144,450	Disease resistance protein (TIR-NBS-LRR class) family
ptc-miR482d-3p	*Arahy.LVW2ZC.1*	2	2,165,965	2,179,064	LRR and NB-ARC domain disease resistance protein
	*Arahy.SLVW9F.1*	12	1,845,409	1,846,752	Disease resistance protein (TIR-NBS-LRR class) family
	*Arahy.Y2F96D.1*	12	2,634,170	2,637,760	LRR and NB-ARC domain disease resistance protein
	*Arahy.KL7N91.1*	12	2,857,891	2,861,052	LRR and NB-ARC domain disease resistance protein
	*Arahy.77RH3X.1*	12	2,840,034	2,841,879	LRR and NB-ARC domain disease resistance protein
	*Arahy.104ZDW.1*	4	126,965,165	126,971,113	Disease resistance protein (TIR-NBS-LRR class) family
	*Arahy.NUHQ9Q.1*	4	126,980,888	126,986,798	Disease resistance protein (TIR-NBS-LRR class) family
	*Arahy.6V6NN7.1*	14	141,400,695	141,406,643	Disease resistance protein (TIR-NBS-LRR class) family
	*Arahy.1VN7JI.1*	14	141,416,418	141,422,328	Disease resistance protein (TIR-NBS-LRR class) family
	*Arahy.CA6E74.1*	12	1,892,963	1,907,959	LRR and NB-ARC domain disease resistance protein
	*Arahy.V6X2E8.1*	12	2,827,095	2,838,123	Disease resistance protein (TIR-NBS-LRR class) family
	*Arahy.8HLD5E.1*	12	2,752,265	2,757,090	LRR and NB-ARC domain disease resistance protein
	*Arahy.I5JPYW.1*	12	2,768,839	2,780,577	LRR and NB-ARC domain disease resistance protein
	*Arahy.ZKR6CY.1*	2	2,041,262	2,044,900	LRR and NB-ARC domain disease resistance protein
	*Arahy.UZFH7Q.1*	14	141,463,237	141,467,397	Disease resistance protein (TIR-NBS-LRR class) family
	*Arahy.REWL7K.1*	4	127,027,707	127,031,867	Disease resistance protein (TIR-NBS-LRR class) family
	*Arahy.51RKDV.1*	12	2,485,257	2,492,769	LRR and NB-ARC domain disease resistance protein
	*Arahy.M55R6K.1*	2	16,662,936	16,665,269	Disease resistance protein (TIR-NBS-LRR class) family
	*Arahy.DLTR3L.1*	12	2,445,028	2,450,508	LRR and NB-ARC domain disease resistance protein
	*Arahy.II44X3.1*	4	127,073,261	127,076,829	Disease resistance protein (TIR-NBS-LRR class) family
	*Arahy.388Y5C.1*	14	141,508,791	141,512,359	Disease resistance protein (TIR-NBS-LRR class) family
	*Arahy.N06FBV.1*	2	1,506,683	1,516,435	LRR and NB-ARC domain disease resistance protein
	*Arahy.15H21N.1*	2	509,289	513,987	LRR and NB-ARC domain disease resistance protein
	*Arahy.K68I1Q.1*	12	2,901,005	2,904,685	LRR and NB-ARC domain disease resistance protein
	*Arahy.YM09LB.1*	2	2,011,567	2,015,247	LRR and NB-ARC domain disease resistance protein
	*Arahy.JUY39I.1*	3	123,658,764	123,662,492	LRR and NB-ARC domain disease resistance protein
	*Arahy.VMD6HC.1*	2	14,047,304	14,048,911	Disease resistance protein (TIR-NBS-LRR class) family
	*Arahy.2HN52Q.1*	12	2,535,411	2,539,172	LRR and NB-ARC domain disease resistance protein
	*Arahy.3TW696.1*	2	2,455,813	2,460,791	LRR and NB-ARC domain disease resistance protein
	*Arahy.7S97YI.1*	12	2,915,861	2,919,511	LRR and NB-ARC domain disease resistance protein
	*Arahy.25Q9K5.1*	12	2,793,908	2,797,486	LRR and NB-ARC domain disease resistance protein
	*Arahy.73AA2K.1*	12	2,922,302	2,926,138	LRR and NB-ARC domain disease resistance protein
	*Arahy.MZFR22.1*	2	1,565,683	1,568,877	LRR and NB-ARC domain disease resistance protein
	*Arahy.U1TKV1.1*	2	1,543,715	1,549,934	LRR and NB-ARC domain disease resistance protein
	*Arahy.0PVT6F.1*	12	2,588,797	2,592,594	LRR and NB-ARC domain disease resistance protein
	*Arahy.3G3XAR.1*	12	3,267,043	3,271,844	Disease resistance protein (TIR-NBS-LRR class) family
	*Arahy.G3725Q.1*	2	2,193,339	2,196,848	LRR and NB-ARC domain disease resistance protein
	*Arahy.83LA0K.1*	12	1,862,247	1,868,553	LRR and NB-ARC domain disease resistance protein
	*Arahy.8LIU0E.1*	12	2,662,012	2,665,677	LRR and NB-ARC domain disease resistance protein
	*Arahy.TKN2M5.1*	12	3,074,253	3,079,786	LRR and NB-ARC domain disease resistance protein
	*Arahy.FXRP5B.1*	2	2,153,742	2,160,389	Disease resistance protein (TIR-NBS-LRR class) family
	*Arahy.IS7D7R.1*	2	1,869,681	1,883,608	LRR and NB-ARC domain disease resistance protein
	*Arahy.ZBPZ8H.1*	12	13,220,251	13,221,241	Disease resistance protein (TIR-NBS-LRR class) family

## Discussion

Groundnut is a protein rich leguminous crop with main source of income in many developing nations [52]. Beyond its nutritious value (oil, protein, sugar, vitamins and minerals), it also plays an important role in sustainable agriculture due to its ability to thrive in marginal soil, withstand drought and fix nitrogen [53]. However, groundnut is susceptible to aflatoxin during both the pre-harvest and post-harvest stages [54]. An integrated management strategy is crucial to minimize the risk of aflatoxin contamination. Aflatoxin are generally secondary metabolites produced by soil-borne saprophytic group of genus *Aspergillus* which affect groundnut and other food commodities. Environmental parameters such as high soil temperature, moisture stress, relative humidity influence the *A. flavus* infection and subsequent aflatoxin accumulation. The lack of genetic resistance in groundnut, along with these environmental factors, limited progress in this direction, makes this trait very complex [55]. At the post-transcriptional level, small non-coding RNAs such as miRNAs have been identified as significant regulators of gene expression [56]. miRNAs are non-coding RNAs that play vital roles in developmental processes and stress responses through negative regulations [57].

In this study, the infected and controlled small RNA libraries of *A. flavus* were prepared to identify the known and novel miRNAs from resistant and susceptible varieties. Among 208 known miRNA, 25 miRNAs were first reported in groundnut by Zhao et al*.* [35], 42 miRNAs were reported by Chi et al. [31] and 66 miRNAs were identified by Zhao et al. [58]. Previous studies reported that known miRNAs are majorly involved in developmental processes, whereas novel miRNAs were the part of species-specific gene regulatory functions [59, 60]. These findings illustrated the conservation of miRNAs, supporting previous studies that reported the presence of over 21 conserved miRNAs among monocots, dicots, and mosses [61]. Due to their conserved nature, these miRNAs exhibit homologous target interactions, resulting in analogous annotations across different species [62].

Some features of miRNAs such as length and GC content were in concordance with the previous studies [63, 64]. The findings of this study unveiled that the average GC content of groundnut miRNA was approximately 51.05%, which closely resembled the GC content of chickpea miRNA (48%) [65]. The known miR families such as miR166, miR167 and miR156 were identified more frequently and abundantly expressed, consistent with previous studies [31, 66, 67]. In the present study, total 186 and 199 variants of known miRNAs were identified from different treatments of resistant and susceptible varieties. The abundance of variants for most of known miRNA families were higher in JL 24 than J 11, and miR166 having more variants than other families. All novel miRNAs were not registered in miRbase which support the evidence to declare them as novel miRNAs. The accurate identification of miRNA targets allows for the inference of their functions. The known and novel miRNAs were searched against Nucleotides, GSS, ESTs and TSA sequence of *Arachis* using the psRNATarget server. The identified targets were annotated by BLAST against the NCBI Nr Database. A total of 1,742 targets were identified, with some miRNAs having more than one targets. Functional annotation and classification showed that 22.8% of the targets were found to be associated with disease resistance proteins such as RPP and TMV resistance proteins, 5.6% with unknown proteins, 3.6% with transcription factors such as TCP4, TCP3, MYB52, MYB 97, and remaining were associated with receptors like serine/threonine protein kinase, mitogen activated protein, growth regulating factor. Interestingly, most miRNAs were predicted to target resistance genes analog. The number of disease resistance proteins-coding genes were the target of miRNAs such as Recognition of *Peronospora parasitica* 13 (RPP) known to encode 820 amino acids which were believed to reside within cytoplasm and function in LRR (Leucine rich repeat) synthesis [68]. Similarly, RFL1 protein which was domain in NBS-LRR [69], TAO1 contributes to disease resistance in response to *Pseudomonas syringae* pathovars of tomato [70], Dominant Suppressor of Camta 3 number 1 (DSC1) which is immune receptor of TIR-NB-LRR [71], leucine-rich repeat receptor-like protein kinase and leaf rust disease-resistance locus receptor-like protein kinase, TMV resistance protein and LRR receptor-like serine/threonine-protein kinase are the putative protein encoded by the targeted genes found in this study. The NBS-LRRs are composed of a nucleotide-binding domain located at the center, which is connected to a leucine-rich repeat (LRR) domain at the C-terminal end. Additionally, there is a variable N-terminal domain that can either be a coiled-coil (CC) domain or a Toll-interleukin-1 receptor (TIR)-like domain [72]. Apart from disease resistance proteins, transcription factors like bHLH155, bHLH19, DUO1, GAMYB, MYB33, MYB52, MYB97, TCP2 were also target of identified miRNAs.

Total 229 miRNAs showed the differential expression in at least one treatment, and it was noted that all novel miRNAs were among the differentially expressed miRNAs. Moreover, it was seen that members of the same miRNA families responded differently to *A. flavus* infection. For example, miRNAs from family miR156 namely aly-miR156d-3p showed upregulation (Log2 FC = 2.51) at 7 DAI in J 11 whereas ath-miR156b-3p was downregulated (Log2 FC = −1.64) at 7 DAI in J 11. Similar trend was previously reported in other crops such as chickpea where miR171 showed differential expression pattern under Ascochyta blight infection [65] and soybean in which miR396 showed differential expression at high cadmium concentration [73]. In response to fungus *Exserohilum turcicum*, the miRNAs namely, zma-miR811, zma-miR829, zma-miR845 and zma-miR408 showed differential expression in maize [74]. Furthermore, it has been reported that the miRNA zma-miR408 plays a regulatory role in gene expression related to the defense response against *Fusarium verticillioides*, the fungus responsible for ear rot, in maize [75]. Total seven miRNAs in J 11 were continuously expressed at 1 DAI, 2 DAI, 3 DAI, and 7 DAI, while ten miRNAs were commonly expressed in JL 24. Similarly, commonly expressed miRNAs (Osa-miR156d, Osa-miR159b, Osa-miR820c, and Osa-miR1876) were identified between susceptible and resistant rice cultivars [76]. The miRNAs, mtr-miR2118 and ptc-miR482d-3p were belongs to miR2118 and miR482 families. The members of these families are known to play major roles in stress response [77]. The targets of mtr-miR2118 were found on chromosome 5, 9, 15 and 19 whereas targets of ptc-miR482d-3p located on chromosome 2, 3, 4, 12 and 14, and all these targets were associated with TIR-NBS-LLR encoding domains. The miR2118 was reported as a member of miR482 superfamily due to sequence similarity [42]. The miRNA families, miR2118 and miR482, specifically targeted the *NBS-LRR* genes, which are crucial components involved in disease resistance mechanisms in plants [42, 78]. Typically, these miRNA families target the conserved P-loop regions within the NBS domain of *NBS-LRR* genes [77]. The miRNAs, aly-miR156d-3p, csi-miR1515a, gma-miR396e, mtr-miR2118, novo-miR-n27, ppe-miR396a and ptc-miR482d-3p were found to be differentially expressed across different inoculation days in J 11 but not in JL 24, highlighted their potential role in defense response against *A. flavus* infection in groundnut. Similarly, a total of 21 differentially expressed miRNAs were identified exclusively in the resistant variety Mp719, while no such miRNAs were found in the susceptible variety Va35, which suggested that these miRNAs might role in conferring resistance to *A. flavus* infection in maize [79].

The functional roles of mtr-miR2118 and ptc-miR482d-3p was based on insilico analysis. Subsequently, the exploration and functional validation of variation within mtr-miR2118 and ptc-miR482d-3, along with their target genes are important. This will identify beneficial alleles for improving trait, while also providing insights into their evolutionary roles and contributions to crop domestication. Moreover, natural variation within miRNA-binding sites could result in significant phenotypic changes. The introduction of in-frame mutations using CRISPR/Cas9 within the miRNA-complementary regions of target genes could decrease miRNA-induced repression, potentially increase resistance to *A. flavus.*

## Conclusion

In this study, we identified 208 known and 27 novel miRNAs in J 11 and JL 24 groundnut varieties. Total 1742 targets were identified for these miRNAs, which were found to encode disease resistant proteins, transcription factor involved in several metabolic pathways, transmembrane receptors, and protein kinase family proteins. There were only two (mtr-miR2118 and ptc-miR482b-3p) differentially expressed miRNAs which expressed at all days after inoculations in both the resistant, (J 11) and susceptible, (JL 24) varieties. Further, the in silico expression analysis revealed the tissue- specific expression of target genes of these two miRNAs. Functional annotation of these genes, showed that the genes were known to be involved in disease resistance mechanism by regulating the expression of various proteins like TIR-NBS-LRR, TMV resistance protein and serine/threonine protein kinase. These targets of miRNAs in resistance against *A. flavus* can be used in the development of markers for groundnut breeding program to enhance resistance against *A. flavus*. Furthermore, the interactions between mtr-miR2118 and ptc-miR482b-3p and their respective target genes can be validated by derepressing the miRNA-complementary regions of the corresponding target genes using CRISPR/Cas9. This approach will generate in-frame mutants, which may enhance resistance against *A. flavus* infection.

## Supplementary Information

Additional file 1.

Additional file 2.

Additional file 3.

Additional file 4.

## Data Availability

The datasets generated and/or analysed during the current study are available in the NCBI repository, BioProject ID PRJNA355201.
