# Supplementary figures and images for: Identification of miRNAs associated with Aspergillus flavus infection and their targets in groundnut (Arachis hypogaea L.)
Source: BMC Plant Biol. 2025 Mar 18;25:345. doi: 10.1186/s12870-025-06322-2 (PMC11917013; doi:10.1186/s12870-025-06322-2)

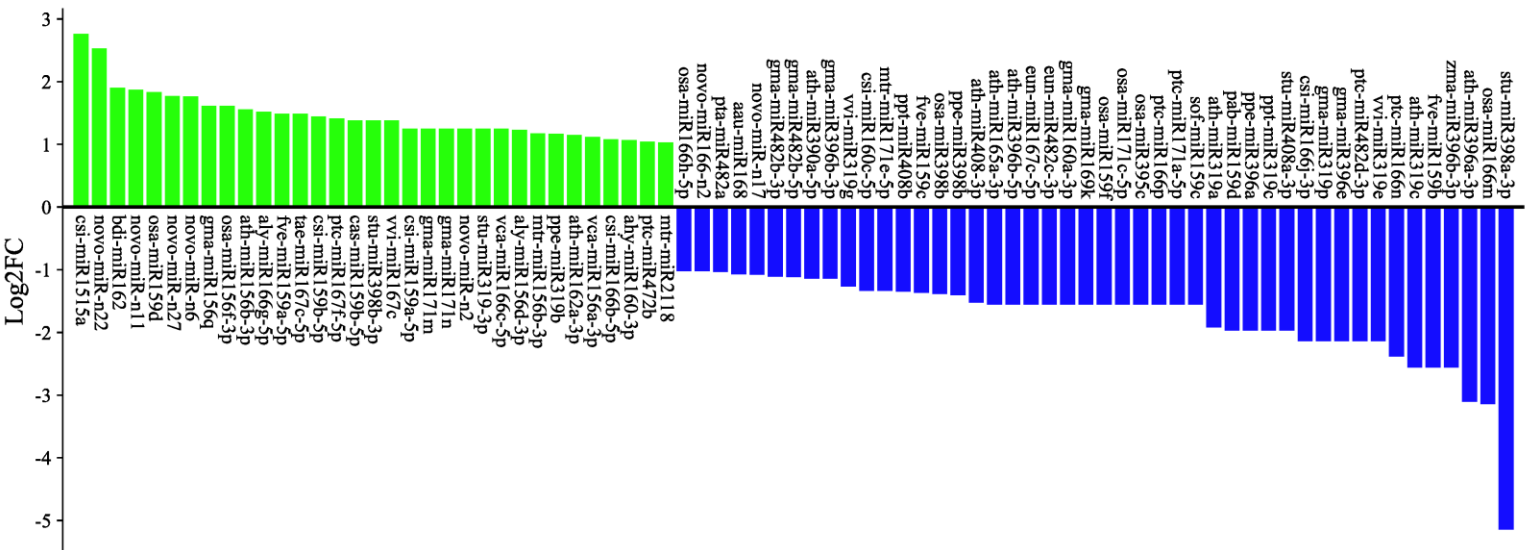

(A)

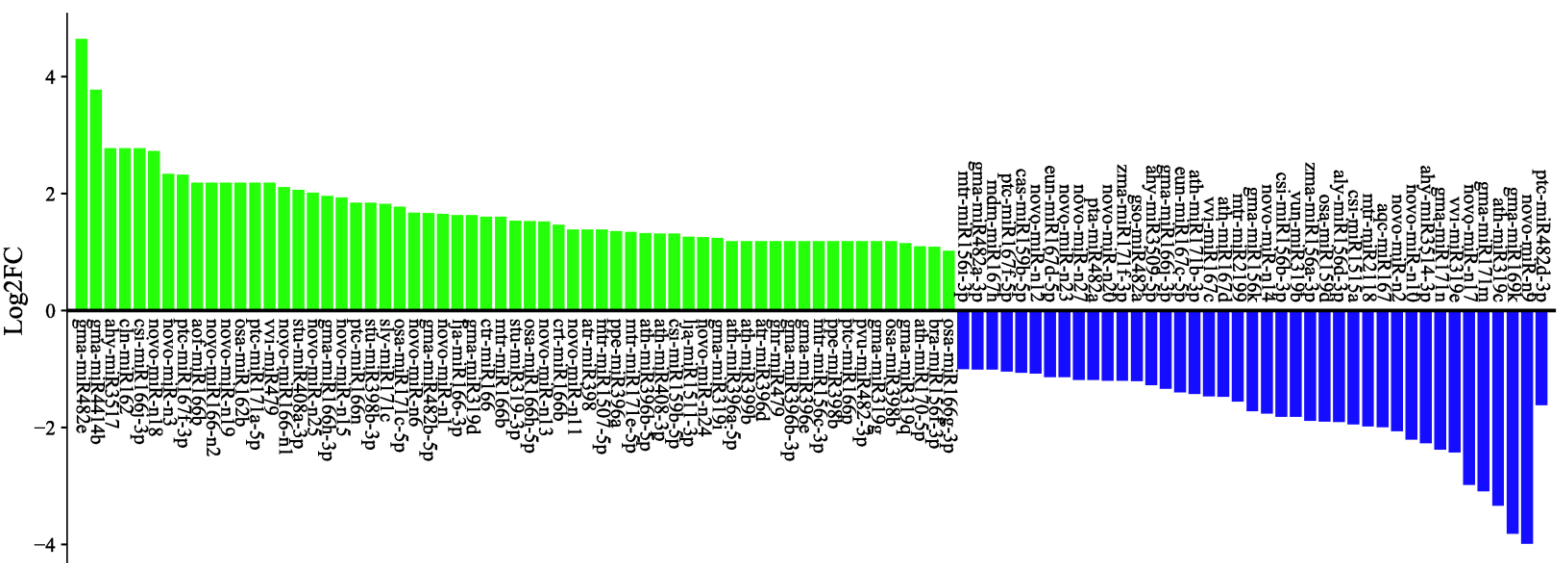

(B)

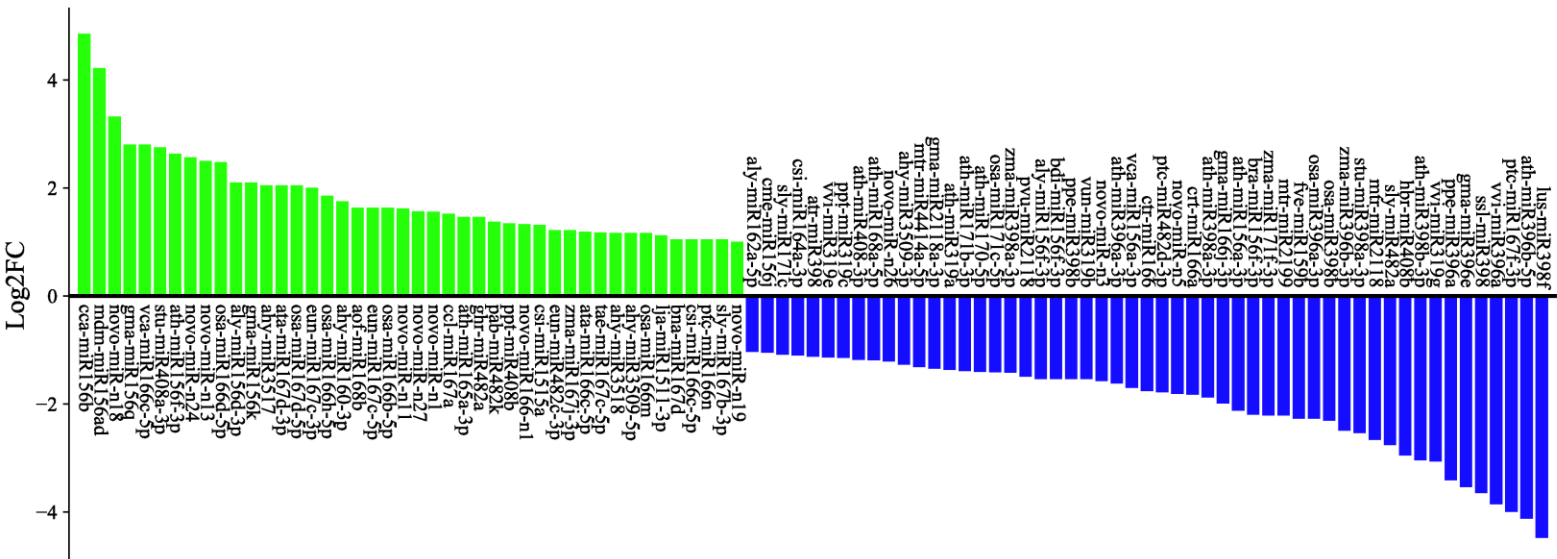

(C)

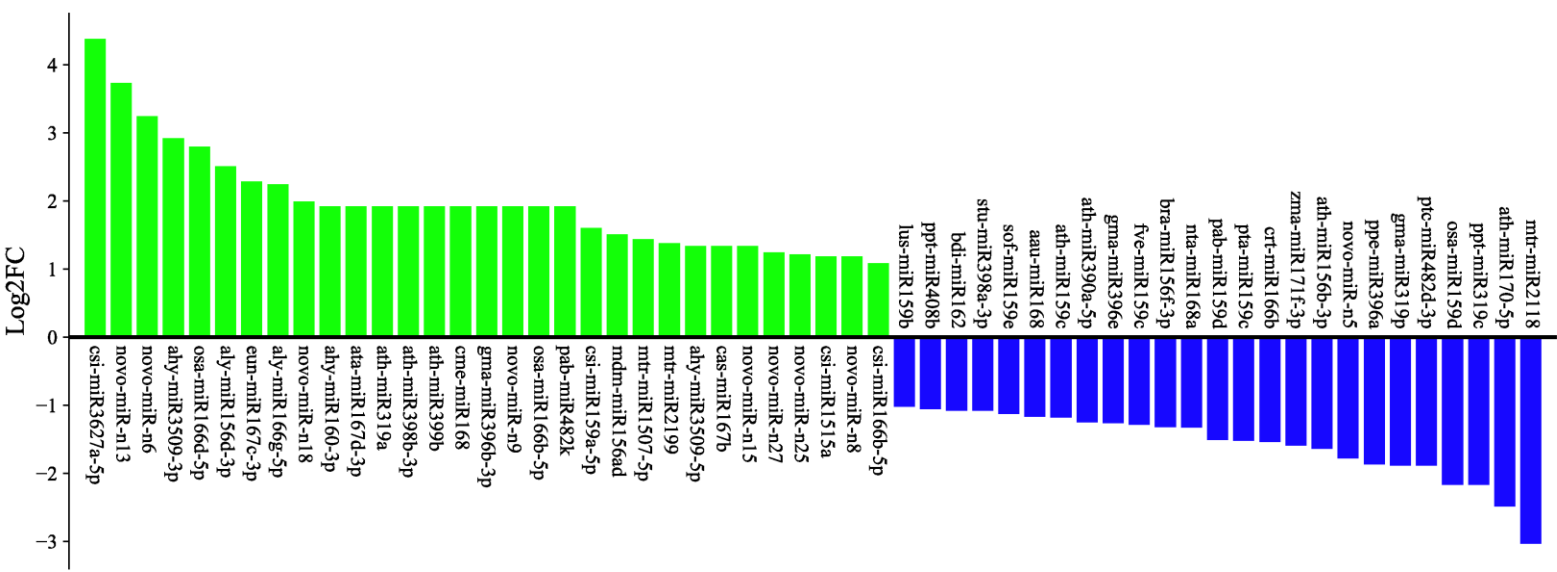

(D)

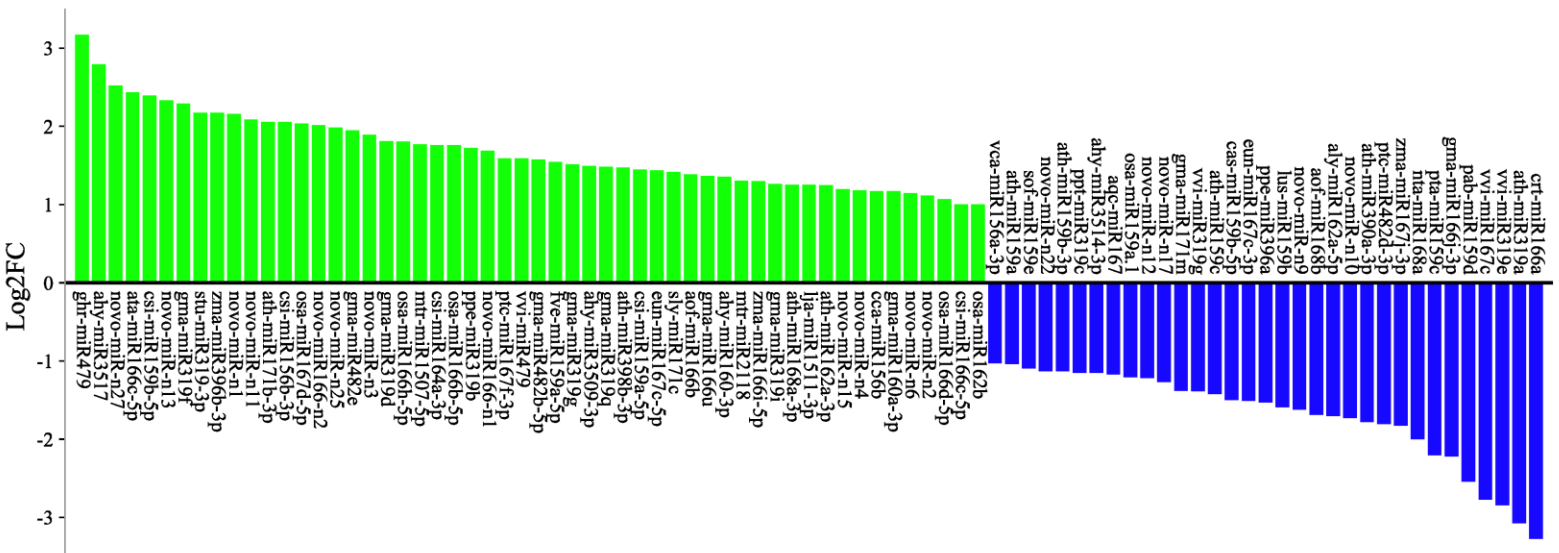

(E)

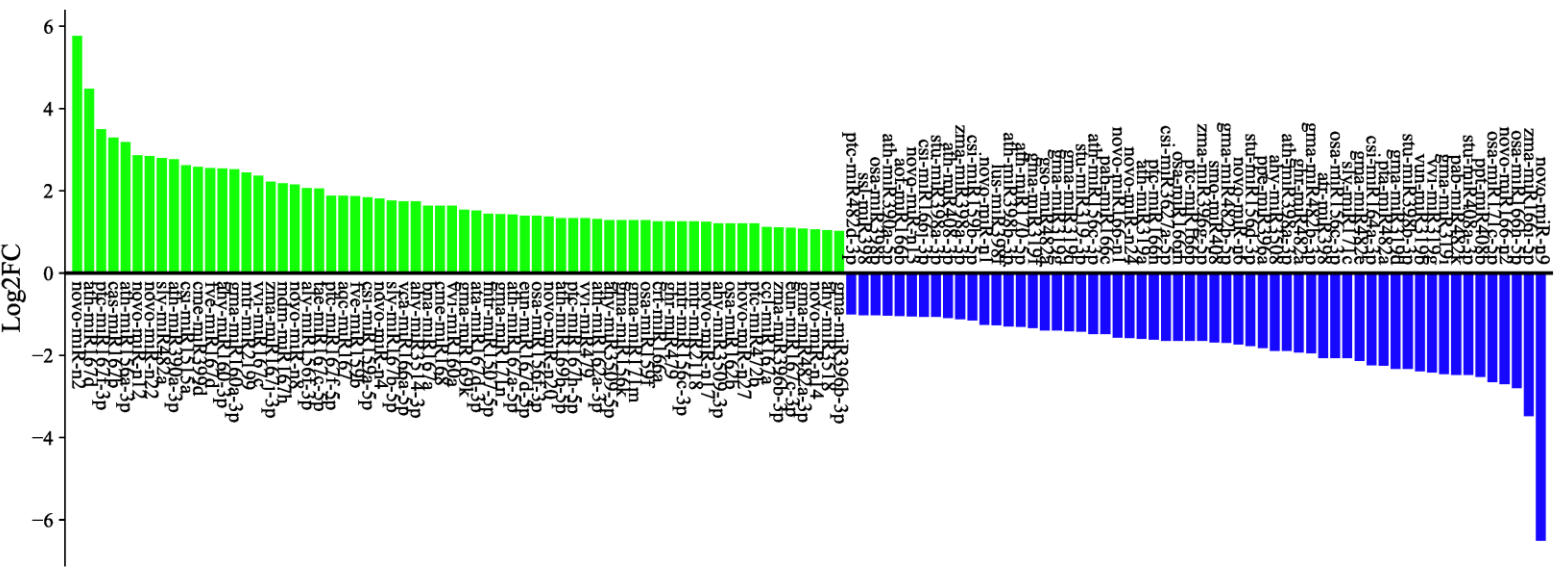

(F)

(G)

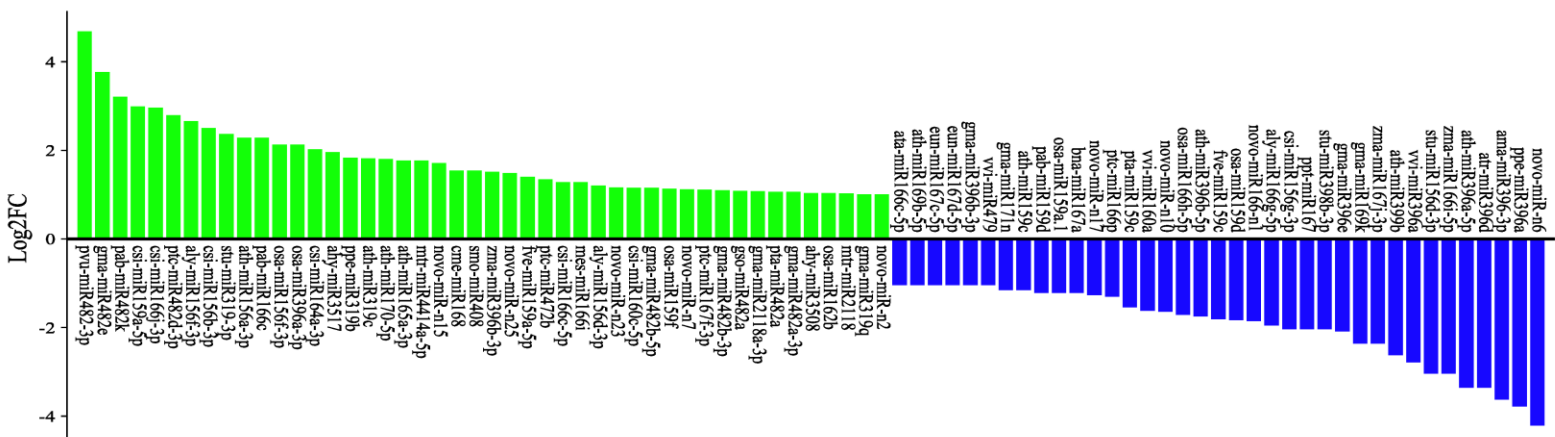

(H)

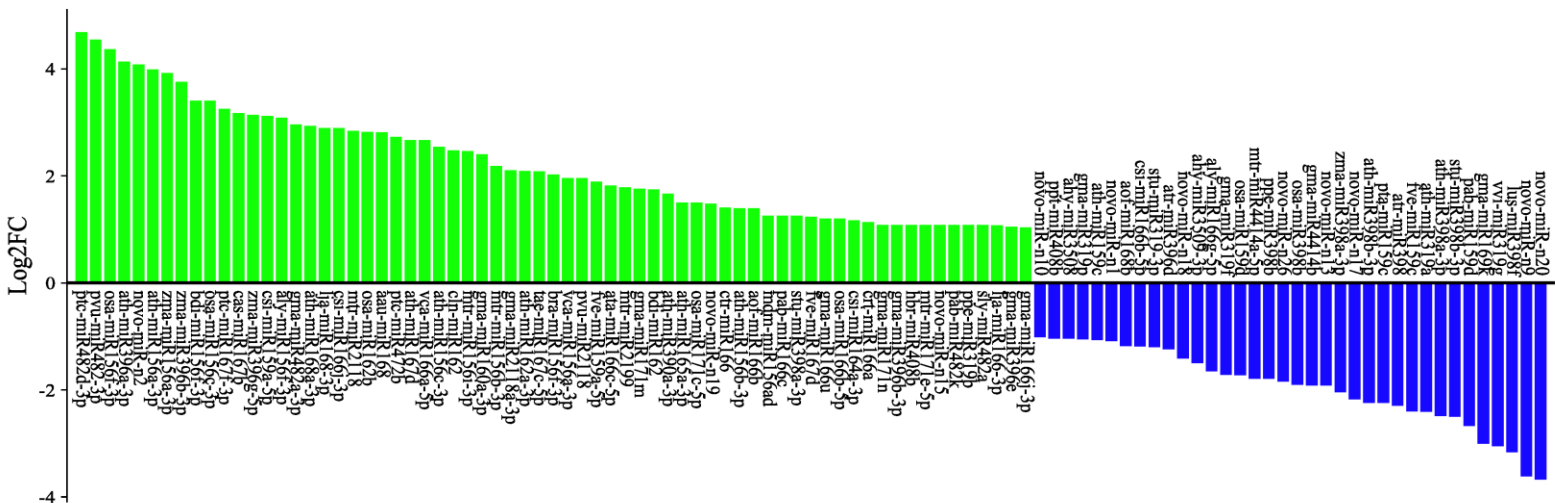

Supplement: Supplementary file 2 — Additional file 2. [file 12870_2025_6322_MOESM2_ESM.pdf]
