# Supplementary material for: Identification of miRNAs associated with Aspergillus flavus infection and their targets in groundnut (Arachis hypogaea L.)
Source: BMC Plant Biol. 2025 Mar 18;25:345. doi: 10.1186/s12870-025-06322-2 (PMC11917013; doi:10.1186/s12870-025-06322-2)
Supplement: Supplementary file 4 — Additional file 4. [file 12870_2025_6322_MOESM4_ESM.pdf]

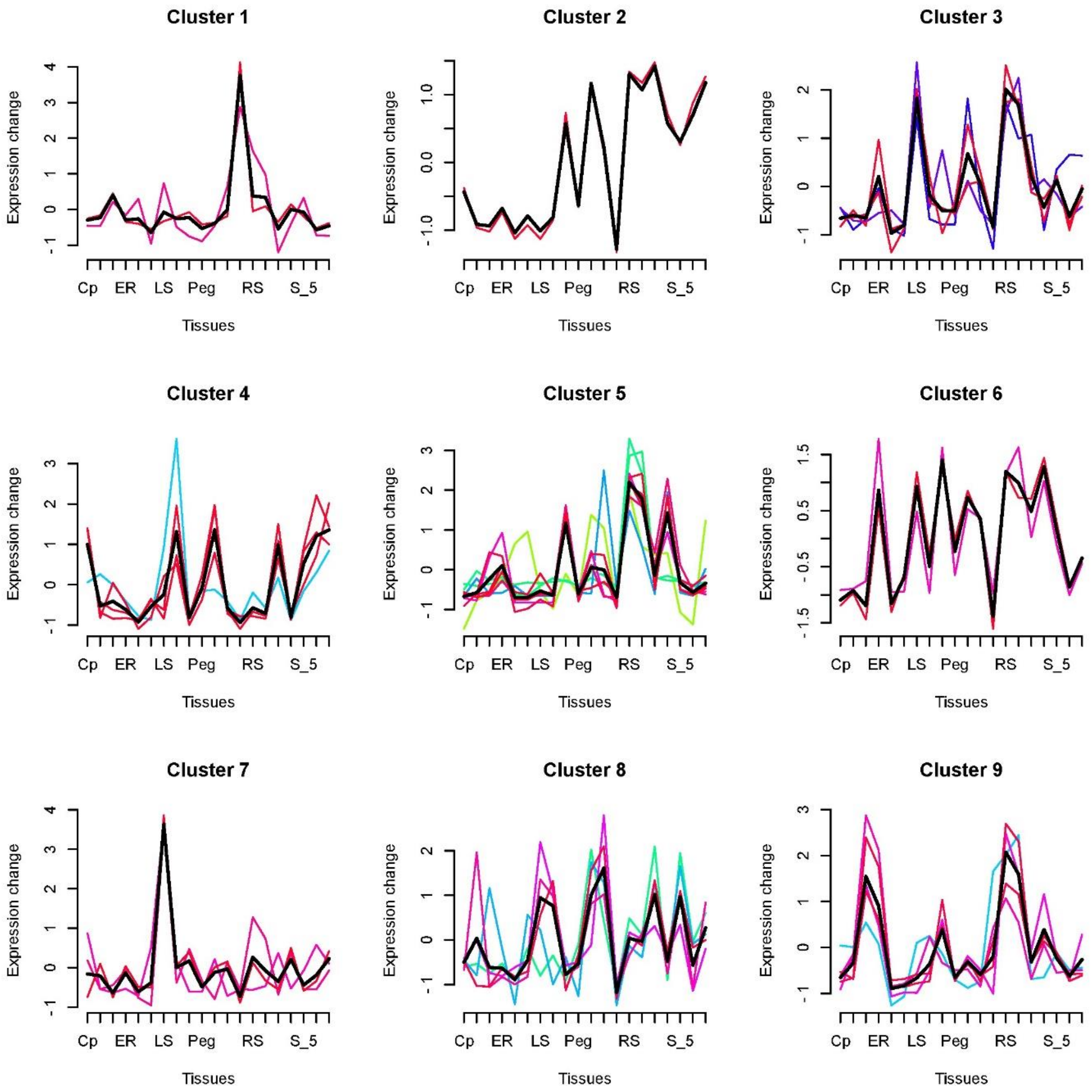

Additional file 4: Total nine clusters depicted the expression trends of targeted 47 genes in 20 different tissues; **Cluster 1:** *Arahy.8HLD5E*, *Arahy.I5JPYW*, *Arahy.N06FBV*, *Arahy.ZKR6CY*; **Cluster 2:** *Arahy.77RH3X*, *Arahy.KL7N91*, *Arahy.Y2F96D*; **Cluster 3:** *Arahy.7J0RKL*, *Arahy.G3725Q*, *Arahy.REWL7K*, *Arahy.SLVW9F*, *Arahy.UZFH7Q*, *Arahy.VF2B86*; **Cluster 4:** *Arahy.CA6E74*, *Arahy.LVW2ZC*, *Arahy.M55R6K*, *Arahy.MZFR22*; **Cluster 5:** *Arahy.5H21N*, *Arahy.2HN52Q*, *Arahy.3G3XAR*, *Arahy.3TW696*, *Arahy.7S97YI*, *Arahy.83LA0K*, *Arahy.FXRP5B*, *Arahy.H8JIAA*, *Arahy.ZBPZ8H*; **Cluster 6:** *Arahy.NUHQ9Q*, *Arahy.04ZDW*, *Arahy.VN7JI*, *Arahy.388Y5C*, *Arahy.6V6NN7*, *Arahy.II44X3*; **Cluster 7:** *Arahy.25Q9K5*, *Arahy.73AA2K*, *Arahy.TKN2M5*, *Arahy.VMD6HC*; **Cluster 8:** *Arahy.51RKDV*, *Arahy.DLTR3L*, *Arahy.HVB0T8*, *Arahy.JUY39I*, *Arahy.V6X2E8*; **Cluster 9:** *Arahy.0PVT6F*, *Arahy.8LIU0E*, *Arahy.IS7D7R*, *Arahy.K68I1Q*, *Arahy.U1TKV1*, *Arahy.YM09LB*; Cp: Coleoptile; Cd: Cotyledon; ER: Emerging radicle; Fr: Flower; IMB: Immature bud; LS: Leaves senescence; LV: Leaves vegetative; PWI: Pod wall immature;

PWM: Pod wall mature; PSS: Pre-soaked seeds; RS: Root seedling; RV: Root vegetative; S_15: Seeds 15; S_25: Seeds 25; S_5: Seeds 5; SS: Shoot seedling; SV: Stem vegetative.
